# Supplementary figures and images for: An integrative taxonomic analysis reveals a new species of lotic Hynobius salamander from Japan
Source: PeerJ. 2018 Jun 21;6:e5084. doi: 10.7717/peerj.5084 (PMC6015758; doi:10.7717/peerj.5084)

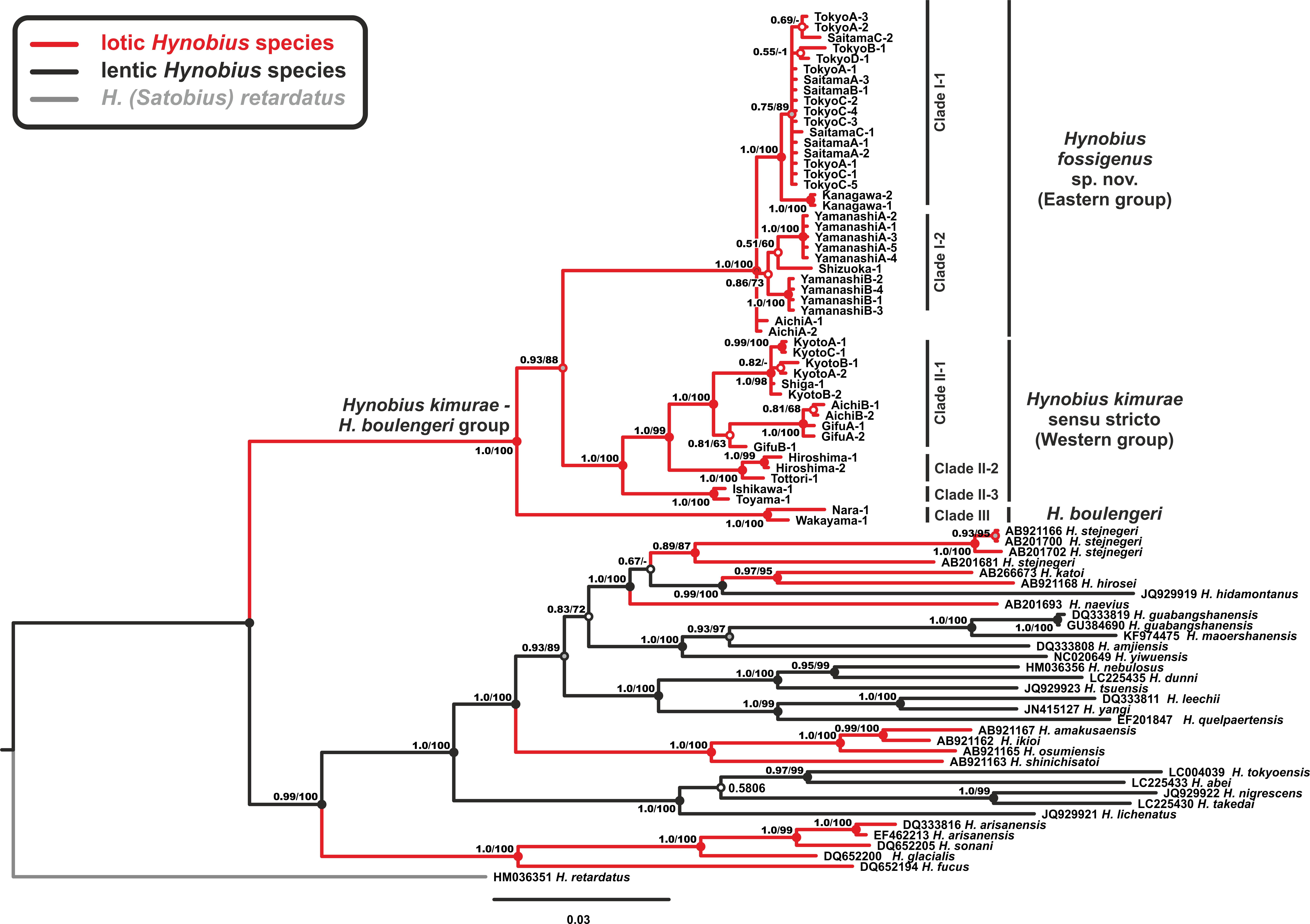

Supplement: Supplemental Information 1 — Values on the branches correspond to BI PP/ML BS, respectively; filled, grey and white circles correspond to well-supported, moderately supported and non-supported nodes, respectively. Red color denotes lotic (stream-breeding) Hynobius, black color denotes lentic (still water-breeding) Hynobius species. Hynobius (Satobius) retardatus is a sister taxon to all other Hynobius and combines features of both lotic and lentic species. For locality information and voucher info see Tables S1 and S3. [file peerj-06-5084-s001.png]

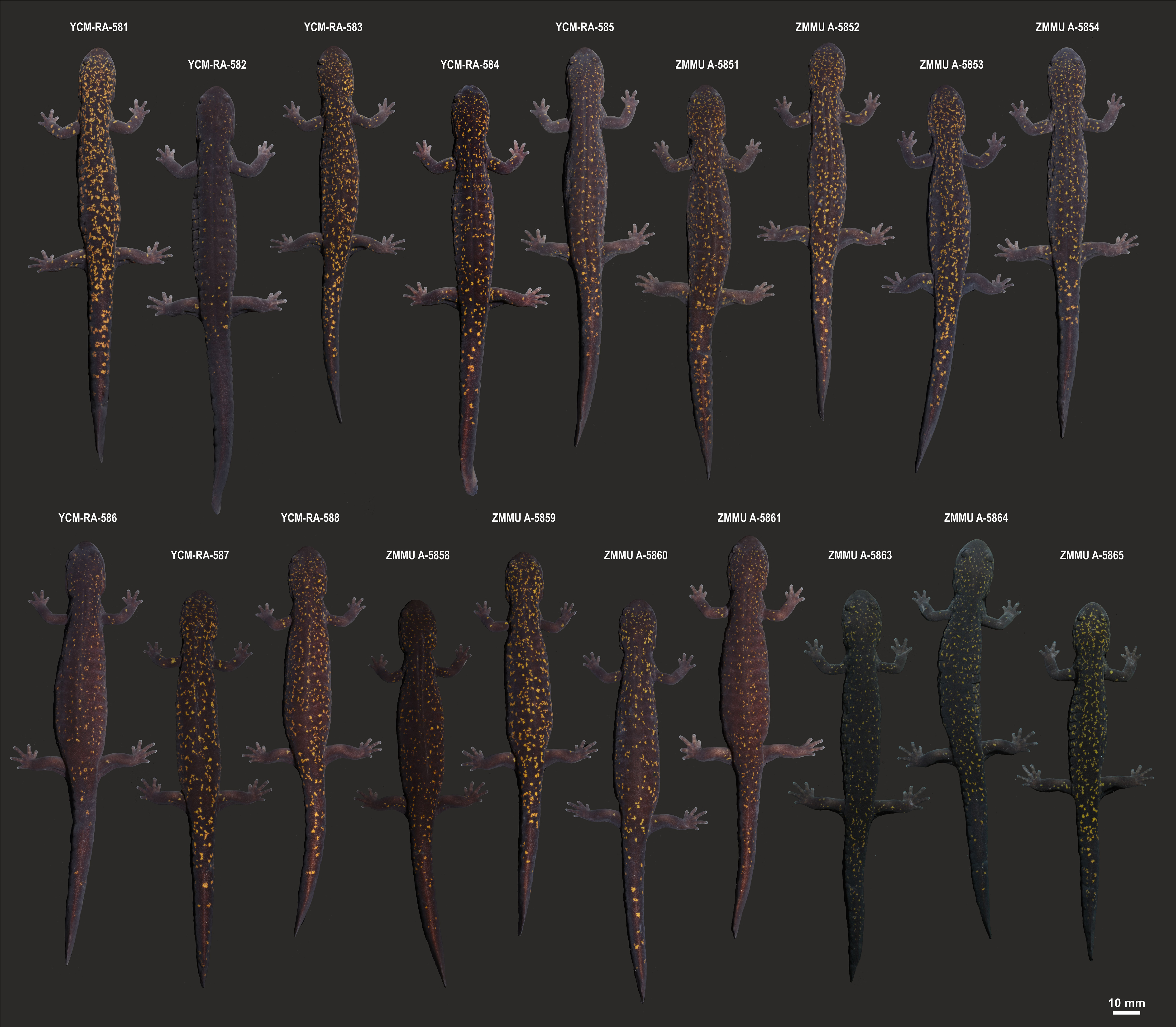

Supplement: Supplemental Information 2 — Upper row and three specimens from the right in the lower row–males; seven specimens from the left in the lower row–females. Scale bar indicates 10 mm. Photos by H. Okamiya and N.A. Poyarkov. [file peerj-06-5084-s002.jpg]
