# Supplementary material for: An integrative taxonomic analysis reveals a new species of lotic Hynobius salamander from Japan
Source: PeerJ. 2018 Jun 21;6:e5084. doi: 10.7717/peerj.5084 (PMC6015758; doi:10.7717/peerj.5084)
Supplement: Supplemental Information 3 — For details on geographic location of examined populations see Table S2. [file peerj-06-5084-s003.docx]

| **№** | **Species** | **Population** | | **Sample ID** | **GenBank accession no.** | | | **Source** |
| --- | --- | --- | --- | --- | --- | --- | --- | --- |
|  |  | **No.** | **name** | **(voucher ID in brackets)** | **16S** | ***cyt b*** | **Rag1** |  |
|  | **Ingroup** |  |  |  |  |  |  |  |
| 1 | *Hynobius fossigenus* **sp. nov.** | **1** | SaitamaA | SaitamaA-1 | MH253618 | MH287387 | MH287353 | This study |
| 2 | (Eastern group) | *"* | *"* | SaitamaA-2 | MH253619 | MH287388 | MH287354 | This study |
| 3 | *"* | *"* | *"* | SaitamaA-3 | MH253620 | MH287389 | na | This study |
| 4 | *"* | **2** | SaitamaB | SaitamaB-1 | MH253621 | MH287390 | MH287355 | This study |
| 5 | *"* | **3** | SaitamaC | SaitamaC-1 | MH253622 | MH287391 | MH287356 | This study |
| 6 | *"* | *"* | *"* | SaitamaC-2 | MH253623 | MH287392 | na | This study |
| 7 | *"* | **4** | TokyoA | TokyoA-1  (ZMMU A-5883) | MH253624 | MH287393 | MH287357 | This study |
| 8 | *"* | *"* | *"* | TokyoA-2  (ZMMU A-5884) | MH253625 | MH287394 | MH287358 | This study |
| 9 | *"* | *"* | *"* | TokyoA-3 | MH253626 | MH287395 | na | This study |
| 10 | *"* | *"* | *"* | TokyoA-4 | MH253635 | na | na | This study |
| 11 | *"* | *"* | *"* | TokyoA-5 | MH253636 | MH287431 | na | This study |
| 12 | *"* | **5** | TokyoB | TokyoB-1 | MH253627 | MH287396 | MH287359 | This study |
| 13 | *"* | **6** | TokyoC | TokyoC-1  (ZMMU A-5866) | MH253628 | MH287397 | MH287360 | This study |
| 14 | *"* | *"* | *"* | TokyoC-2  (ZMMU A-5867) | MH253629 | MH287398 | MH287361 | This study |
| 15 | *"* | *"* | *"* | TokyoC-3  (ZMMU A-5868) | MH253630 | MH287399 | na | This study |
| 16 | *"* | *"* | *"* | TokyoC-4  (ZMMU A-5869) | MH253631 | MH287400 | na | This study |
| 17 | *"* | *"* | *"* | TokyoC-5  (ZMMU A-5870) | MH253632 | MH287401 | na | This study |
| 18 | *"* | **7** | TokyoD | TokyoD-1 | na | MH287432 | na | This study |
| 19 | *"* | **8** | Kanagawa | Kanagawa-1 | MH253633 | MH287402 | MH287362 | This study |
| 20 | *"* | *"* | *"* | Kanagawa-2 | MH253634 | MH287403 | MH287363 | This study |
| 21 | *"* | **9** | YamanashiA | YamanashiA-1 | MH253637 | MH287404 | MH287364 | This study |
| 22 | *"* | *"* | *"* | YamanashiA-2 | MH253638 | MH287405 | MH287365 | This study |
| 23 | *"* | *"* | *"* | YamanashiA-3 | MH253639 | MH287406 | na | This study |
| 24 | *"* | *"* | *"* | YamanashiA-4 | MH253640 | MH287407 | na | This study |
| 25 | *"* | *"* | *"* | YamanashiA-5 | MH253641 | MH287408 | na | This study |
| 26 | *"* | **10** | YamanashiB | YamanashiB-1 | MH253642 | MH287409 | MH287366 | This study |
| 27 | *"* | *"* | *"* | YamanashiB-2 | MH253643 | MH287410 | MH287367 | This study |
| 28 | *"* | *"* | *"* | YamanashiB-3 | MH253644 | MH287411 | na | This study |
| 29 | *"* | *"* | *"* | YamanashiB-4 | MH253645 | MH287412 | na | This study |
| 30 | *"* | **11** | Shizuoka | Shizuoka-1 | MH253646 | MH287413 | MH287368 | This study |
| 31 | *"* | **12** | AichiA | AichiA-1 | MH253647 | MH287414 | MH287369 | This study |
| 32 | *"* | *"* | *"* | AichiA-2 | MH253648 | MH287415 | MH287370 | This study |
| 33 | *Hynobius kimurae* s. str. | **13** | AichiB | AichiB-1 | MH253651 | MH287418 | MH287371 | This study |
| 34 | (Western group) | *"* | *"* | AichiB-2 | MH253652 | MH287419 | MH287372 | This study |
| 35 | *"* | **14** | GifuA | GifuA-1 | MH253653 | MH287420 | MH287373 | This study |
| 36 | *"* | *"* | *"* | GifuA-2 | MH253654 | MH287421 | MH287374 | This study |
| 37 | *"* | **15** | GifuB | GifuB-1 | MH253655 | MH287422 | MH287375 | This study |
| 38 | *"* | **16** | Toyama | Toyama-1 | MH253649 | MH287416 | MH287382 | This study |
| 39 | *"* | **17** | Ishikawa | Ishikawa-1 | MH253650 | MH287417 | MH287381 | This study |
| 40 | *"* | **–** | Shiga | Shiga-1 | na | AB266674 | na | *Matsui et al. (2007)* |
| 41 | *"* | **18** | KyotoA | KyotoA-1 | MH253657 | MH287423 | MH287376 | This study |
| 42 | *"* | *"* | *"* | KyotoA-2 | MH253658 | MH287424 | MH287377 | This study |
| 43 | *"* | *"* | *"* | KyotoA-3 | na | na | KJ715369 | *Chen et al. (2015)* |
| 44 | *"* | **19** | KyotoB | KyotoB-1  (ZMMU A-5908) | MH253659 | MH287425 | MH287378 | This study |
| 45 | *"* | *"* | *"* | KyotoB-2  (ZMMU A-5909) | MH253660 | MH287426 | MH287379 | This study |
| 46 | *"* |  | KyotoC | KyotoC-1 | MH253661 | MH287433 | MH287380 | This study |
| 47 | *"* | *"* | *"* | KyotoC-2 | MH253656 | na | na | This study |
| 48 | *"* | **–** | KyotoD | KyotoD-1 | AB201705 | na | na | *Tominaga et al. (2006)* |
| 49 | *"* | **20** | HyogoA | HyogoA-1 | MH253662 | na | na | This study |
| 50 | *"* | *"* | *"* | HyogoA-2 | MH253663 | na | na | This study |
| 51 | *"* | **21** | HyogoB | HyogoB-1 | MH253664 | na | na | This study |
| 52 | *"* | **22** | Tottori | Tottori-1 | MH253665 | MH287427 | MH287383 | This study |
| 53 | *"* | **23** | Hiroshima | Hiroshima-1 | MH253666 | MH287428 | MH287384 | This study |
| 54 | *"* | *"* | *"* | Hiroshima-2 | MH253667 | MH287429 | MH287385 | This study |
| 55 | *H. boulengeri* | **24** | Wakayama | Wakayama-1 | MH253668 | MH287430 | MH287386 | This study |
| 56 | *"* | – | Nara | Nara-1 | AB201706 | AB266675 | na | *Tominaga et al. (2006)* |
|  | **Outgroup** |  |  |  |  |  |  |  |
| 57 | *H. guabangshanensis* | – | – | – | GU384690 | GU384690 | na | – |
| 58 | *H. chinensis* | – | – | – | DQ333819 | DQ333819 | na | *Zhang et al. (2006)* |
| 59 | *H. maoershanensis* | – | – | – | KF974475 | KF974475 | na | *Huang et al. (2016)* |
| 60 | *H. amjiensis* | – | – | – | DQ333808 | DQ333808 | na | *Zhang et al. (2006)* |
| 61 | *H. yiwuensis* | – | – | – | NC020649 | NC020649 | na | *Zheng et al. (2011)* |
| 62 | *H. stejnegeri* | – | – | – | AB201702 | AB201702 | na | *Tominaga et al. (2006)* |
| 63 |  | – | – | – | AB201700 | AB201700 | na | *Tominaga et al. (2006)* |
| 64 |  | – | – | – | AB201681 | AB201681 | na | *Tominaga et al. (2006)* |
| 65 | *H. hidamontanus* | – | – | – | JQ929919 | JQ929919 | na | *Zheng et al. (2012)* |
| 66 | *H. naevius* | – | – | – | AB201693 | AB201693 | na | *Tominaga et al. (2006)* |
| 67 | *H. leechii* | – | – | – | DQ333811 | DQ333811 | na | *Zhang et al. (2006)* |
| 68 | *H. yangi* | – | – | – | JN415127 | JN415127 | na | *Lee et al. (2011)* |
| 69 | *H. quelpaertensis* | – | – | – | EF201847 | EF201847 | na | – |
| 70 | *H. tsuensis* | – | – | – | JQ929923 | JQ929923 | na | *Zheng et al. (2012)* |
| 71 | *H. nebulosus* | – | – | – | HM036356 | HM036356 | na | *Zheng et al. (2011)* |
| 72 | *H. nigrescens* | – | – | – | JQ929922 | JQ929922 | na | *Zheng et al. (2012)* |
| 73 | *H. lichenatus* | – | – | – | JQ929921 | JQ929921 | na | *Zheng et al. (2012)* |
| 74 | *H. tokyoensis* | – | – | – | HM036357 | LC004039 | na | *Zheng et al. (2011)*; *Sugawara et al. (2016)* |
| 75 | *H. arisanensis* | – | – | – | EF462213 | EF462213 | na | – |
| 76 | *H. formosanus* | – | – | – | DQ333816 | DQ333816 | na | *Zhang et al. (2006)* |
| 77 | *H. retardatus* | – | – | – | HM036351 | HM036351 | na | *Zheng et al. (2011)* |
