# Supplementary material for: An integrative taxonomic analysis reveals a new species of lotic Hynobius salamander from Japan
Source: PeerJ. 2018 Jun 21;6:e5084. doi: 10.7717/peerj.5084 (PMC6015758; doi:10.7717/peerj.5084)
Supplement: Supplemental Information 4 — Population numbers and names correspond to those in Table S1 and Fig. 1. [file peerj-06-5084-s004.docx]

| **Pop. No.** | **Species** | **Longitude** | **Latitude** | **Pop. Name** | **Prefecture** | **City** | **Locality** |
| --- | --- | --- | --- | --- | --- | --- | --- |
| 1 | *Hynobius fossigenus* **sp. nov.** | 138.89 | 36.07 | SaitamaA | Saitama | Ogano | Fujikura |
| 2 | (Eastern group) | 138.87 | 36.00 | SaitamaB | Saitama | Ogano | Ryougami |
| 3 |  | 139.10 | 35.90 | SaitamaC | Saitama | Chichibu | Urayama |
| 4 |  | 139.15 | 35.85 | TokyoA | Tokyo | Okutama | Ootaba |
| 5 |  | 139.14 | 35.77 | TokyoB | Tokyo | Oume | Mt. Mitake |
| 6 |  | 139.17 | 35.78 | TokyoC | Tokyo | Hinode | Mt. Hinode |
| 7 |  | 139.03 | 35.74 | TokyoD | Tokyo | Hinohara | Kazuma |
| 8 |  | 139.09 | 35.48 | Kanagawa | Kanagawa | Yamakita | Kurokura |
| 9 |  | 138.51 | 35.33 | YamanashiA | Yamanashi | Nanbu | Kamisano |
| 10 |  | 138.47 | 35.18 | YamanashiB | Yamanashi | Nanbu | Fukushi |
| 11 |  | 137.92 | 35.15 | Shizuoka | Shizuoka | Hamamatsu | Yamazumi |
| 12 |  | 137.72 | 35.22 | AichiA | Aichi | Toyone | Misawa |
| 13 | *H. kimurae* s. str. | 137.30 | 35.28 | AichiB | Aichi | Toyota | Ohgazore |
| 14 | (Western group) | 137.36 | 35.51 | GifuA | Gifu | Nakatsugawa | Mt. Kasagi |
| 15 |  | na | na | GifuB | Gifu | na | na |
| 16 |  | na | na | Toyama | Toyama | na | na |
| 17 |  | 136.82 | 36.79 | Ishikawa | Ishikawa | Houdatsushimizu | Mt. Houdatsu |
| 18 |  | 135.83 | 35.06 | KyotoA | Kyoto | Kyoto | Mt. Hiei |
| 19 |  | 135.74 | 35.14 | KyotoB | Kyoto | Kyoto | Kumogahata |
| 20 |  | 134.92 | 35.21 | HyogoA | Hyogo | Tanba | Aogakicho |
| 21 |  | 134.61 | 35.46 | HyogoB | Hyogo | Mikata | Muraoka |
| 22 |  | na | na | Tottori | Tottori | na | na |
| 23 |  | 132.18 | 34.59 | Hiroshima | Hiroshima | Akiohta | Mt. Uchiguro |
| 24 | *H. boulengeri* | 135.62 | 33.96 | Wakayama | Wakayama | Tanabe | Ryujin |
