# Supplementary material for: An integrative taxonomic analysis reveals a new species of lotic Hynobius salamander from Japan
Source: PeerJ. 2018 Jun 21;6:e5084. doi: 10.7717/peerj.5084 (PMC6015758; doi:10.7717/peerj.5084)
Supplement: Supplemental Information 5 [file peerj-06-5084-s005.docx]

| **No.** | **Species** | **GenBank accession no.** | **Source** |
| --- | --- | --- | --- |
|  |  | ***cyt b*** |  |
|  | **Outgroup** |  |  |
| 1 | *Hynobius katoi* | AB266673 | *Matsui et al. (2007)* |
| 2 | *H. hirosei* | AB921168 | *Nishikawa & Matsui (2014)* |
| 3 | *H. dunni* | LC225435 | *Matsui et al. (2017a)* |
| 4 | *H. stejnegeri* | AB921166 | *Nishikawa & Matsui (2014)* |
| 5 | *H. amakusaensis* | AB921167 | *Nishikawa & Matsui (2014)* |
| 6 | *H. ikioi* | AB921162 | *Nishikawa & Matsui (2014)* |
| 7 | *H. osumiensis* | AB921165 | *Nishikawa & Matsui (2014)* |
| 8 | *H. shinichisatoi* | AB921163 | *Nishikawa & Matsui (2014)* |
| 9 | *H. abei* | LC225433 | *Matsui et al. (2017a)* |
| 10 | *H. takedai* | LC225430 | *Matsui et al. (2017a)* |
| 11 | *H. sonani* | DQ652205 | *Lai & Lue (2008)* |
| 12 | *H. glacialis* | DQ652200 | *Lai & Lue (2008)* |
| 13 | *H. fucus* | DQ652194 | *Lai & Lue (2008)* |
