# Supplementary material for: An integrative taxonomic analysis reveals a new species of lotic Hynobius salamander from Japan
Source: PeerJ. 2018 Jun 21;6:e5084. doi: 10.7717/peerj.5084 (PMC6015758; doi:10.7717/peerj.5084)
Supplement: Supplemental Information 6 — Uncorrected p-distances (percentage) for 16S rRNA (below diagonal) and cyt b (above diagonal) sequences of the Hynobius kimurae–H. boulengeri species complex members and other Hynobius species included in phylogenetic analyses. Within-clade genetic distances for 16S rRNA/cyt b genes are shown on the diagonal in bold. [file peerj-06-5084-s006.docx]

| **No.** | **Lineage** | **1** | **2** | **3** | **4** | **5** | **6** | **7** | **8** | **9** | **10** | **11** | **12** | **13** | **14** | **15** | **16** | **17** | **18** | **19** | **20** | **21** | **22** |
| --- | --- | --- | --- | --- | --- | --- | --- | --- | --- | --- | --- | --- | --- | --- | --- | --- | --- | --- | --- | --- | --- | --- | --- |
| **1** | *H. fossigenus* **sp. nov.** ( I-1) | **0.05/0.35** | 1.30 | 6.24 | 6.11 | 6.30 | 6.46 | 11.97 | 14.24 | 13.85 | 12.70 | 14.35 | 13.81 | 12.40 | 13.19 | 13.04 | 12.15 | 10.74 | 14.01 | 14.25 | 13.20 | 12.84 | 12.20 |
| **2** | *H. fossigenus* **sp. nov.** (I-2) | 0.53 | **0.27/0.71** | 6.09 | 5.98 | 6.59 | 6.27 | 12.07 | 14.14 | 13.83 | 12.55 | 13.72 | 13.12 | 11.83 | 13.14 | 12.83 | 12.00 | 10.54 | 13.95 | 14.27 | 13.24 | 13.00 | 12.23 |
| **3** | *H. kimurae* s. str. (II-1) | 2.85 | 2.91 | **0.61/1.86** | 4.74 | 5.11 | 6.45 | 12.28 | 13.73 | 12.66 | 13.79 | 14.88 | 14.14 | 12.68 | 13.45 | 12.36 | 11.68 | 12.06 | 12.96 | 13.54 | 13.30 | 13.32 | 11.40 |
| **4** | *H. kimurae* s. str. (II-2) | 2.77 | 2.84 | 1.02 | **0.41/0.83** | 5.10 | 6.65 | 12.68 | 14.53 | 13.84 | 13.77 | 14.88 | 15.70 | 12.88 | 14.39 | 13.71 | 13.22 | 12.53 | 14.46 | 15.15 | 14.05 | 13.71 | 12.47 |
| **5** | *H. kimurae* s. str. (II-3) | 2.39 | 2.45 | 1.15 | 1.07 | **0.23/0.41** | 6.20 | 13.72 | 14.67 | 13.84 | 14.46 | 15.08 | 13.22 | 13.43 | 13.84 | 13.84 | 13.64 | 12.60 | 13.84 | 16.12 | 14.26 | 13.64 | 12.60 |
| **6** | *H. boulengeri* (III) | 2.96 | 2.93 | 1.69 | 2.26 | 1.95 | **0.46/1.03** | 11.96 | 13.22 | 13.02 | 13.12 | 13.12 | 13.22 | 12.29 | 13.12 | 12.91 | 12.19 | 12.19 | 13.33 | 14.77 | 14.15 | 12.60 | 13.02 |
| **7** | *H. stejnegeri* | 5.49 | 5.56 | 4.62 | 4.79 | 4.25 | 4.83 | **na** | 11.96 | 12.50 | 11.14 | 12.50 | 14.13 | 11.41 | 11.14 | 10.60 | 13.86 | 11.96 | 11.96 | 14.40 | 13.04 | 14.13 | 11.96 |
| **8** | *H. naevius* | 5.95 | 6.02 | 5.73 | 6.17 | 5.63 | 5.52 | 3.68 | **na** | 10.33 | 10.54 | 11.78 | 11.98 | 11.57 | 12.60 | 11.57 | 12.40 | 11.36 | 11.78 | 11.16 | 13.02 | 13.64 | 15.50 |
| **9** | *H. hidamontanus* | 5.03 | 5.10 | 4.16 | 4.33 | 4.02 | 4.37 | 2.99 | 3.91 | **na** | 13.02 | 13.64 | 13.84 | 12.60 | 12.60 | 11.78 | 12.81 | 10.74 | 13.22 | 12.60 | 13.02 | 16.53 | 14.67 |
| **10** | *H. guabangshanensis* | 6.18 | 6.25 | 4.62 | 4.79 | 4.71 | 5.75 | 3.91 | 4.37 | 3.68 | **na** | 6.20 | 10.33 | 10.95 | 11.57 | 11.16 | 11.36 | 10.74 | 13.22 | 13.22 | 13.02 | 14.46 | 14.26 |
| **11** | *H. maoershanensis* | 6.64 | 6.71 | 5.08 | 5.25 | 5.17 | 6.21 | 3.91 | 3.91 | 3.68 | 0.92 | **na** | 10.74 | 9.92 | 13.22 | 12.60 | 12.40 | 12.19 | 14.05 | 13.84 | 13.84 | 16.32 | 15.70 |
| **12** | *H. amjiensis* | 5.26 | 5.33 | 4.16 | 4.33 | 4.37 | 4.83 | 2.53 | 3.91 | 2.76 | 2.30 | 2.30 | **na** | 9.71 | 12.19 | 11.98 | 13.43 | 11.98 | 15.08 | 13.43 | 11.57 | 15.08 | 13.43 |
| **13** | *H. yiwuensis* | 4.80 | 4.87 | 3.70 | 3.87 | 4.02 | 4.14 | 2.99 | 3.91 | 2.53 | 2.30 | 2.30 | 1.15 | **na** | 10.54 | 9.92 | 13.64 | 10.95 | 13.02 | 11.16 | 10.74 | 13.64 | 12.19 |
| **14** | *H. leechii* | 5.72 | 5.82 | 4.58 | 5.02 | 5.17 | 5.06 | 5.06 | 5.06 | 4.37 | 5.29 | 5.29 | 3.68 | 3.45 | **na** | 6.82 | 11.78 | 10.74 | 13.02 | 15.08 | 12.81 | 14.46 | 13.43 |
| **15** | *H. yangi* | 5.95 | 5.86 | 4.81 | 5.25 | 5.40 | 5.06 | 4.37 | 4.83 | 3.91 | 4.60 | 4.60 | 2.99 | 3.22 | 1.38 | **na** | 9.92 | 9.71 | 13.22 | 12.60 | 11.57 | 14.88 | 13.84 |
| **16** | *H. tsuensis* | 5.03 | 5.10 | 3.89 | 4.33 | 4.02 | 3.91 | 2.53 | 4.14 | 2.53 | 3.91 | 3.91 | 2.99 | 2.76 | 3.45 | 2.76 | **na** | 8.06 | 14.05 | 13.02 | 13.22 | 15.08 | 14.46 |
| **17** | *H. nebulosus* | 5.26 | 5.33 | 4.20 | 4.41 | 4.02 | 4.60 | 3.68 | 4.37 | 2.76 | 2.99 | 2.99 | 3.22 | 3.22 | 3.91 | 3.22 | 2.07 | **na** | 13.64 | 12.81 | 13.43 | 14.46 | 13.43 |
| **18** | *H. nigrescens* | 6.18 | 6.05 | 5.54 | 5.75 | 5.29 | 5.29 | 4.60 | 5.75 | 5.06 | 5.06 | 5.06 | 4.37 | 4.83 | 6.90 | 6.21 | 4.60 | 4.83 | **na** | 10.54 | 12.40 | 14.05 | 14.46 |
| **19** | *H. lichenatus* | 6.18 | 6.25 | 5.12 | 5.71 | 5.29 | 4.83 | 4.60 | 4.83 | 5.06 | 5.29 | 5.29 | 4.14 | 4.60 | 5.75 | 5.52 | 4.14 | 4.60 | 3.45 | **na** | 11.36 | 14.26 | 14.88 |
| **20** | *H. tokyoensis* | 5.49 | 5.56 | 5.00 | 5.10 | 4.60 | 5.06 | 4.14 | 5.06 | 4.83 | 5.75 | 5.75 | 4.83 | 5.06 | 5.98 | 5.98 | 4.37 | 4.37 | 4.83 | 4.83 | **na** | 13.02 | 14.46 |
| **21** | *H. arisanensis* | 5.28 | 5.15 | 4.88 | 5.03 | 4.72 | 3.92 | 5.07 | 5.99 | 4.38 | 5.53 | 5.99 | 5.07 | 4.84 | 5.99 | 5.30 | 3.92 | 5.07 | 5.76 | 5.76 | 6.45 | **na** | 11.78 |
| **22** | *H. retardatus* | 4.11 | 4.18 | 2.97 | 3.41 | 3.10 | 2.53 | 4.60 | 5.06 | 3.68 | 4.37 | 4.83 | 3.91 | 3.22 | 4.14 | 4.37 | 2.99 | 3.91 | 5.52 | 5.06 | 5.29 | 4.15 | **na** |
