# Supplementary material for: An integrative taxonomic analysis reveals a new species of lotic Hynobius salamander from Japan
Source: PeerJ. 2018 Jun 21;6:e5084. doi: 10.7717/peerj.5084 (PMC6015758; doi:10.7717/peerj.5084)
Supplement: Supplemental Information 7 — Ranges are shown in parentheses. For character abbreviations, refer to Supplemental Information 4. N–sample size. Star (*) indicates that Tukey-Kramer test (for SVL) or Kruskal-Wallis test (other characters) show significant differences (p<0.05) for comparisons between Hynobius fossigenus sp. nov. and H. kimurae s. str.; double star (**) indicates Kruskal-Wallis test showing significant differences (p<0.05) for comparisons between Hynobius fossigenus sp. nov. and H. boulengeri. [file peerj-06-5084-s007.docx]

| **Species** | *Hynobius fossigenus* s**p. nov.** (N=18) | | *H. kimurae* s. str.  (N=9) | | *H. boulengeri*  (N=3) | |
| --- | --- | --- | --- | --- | --- | --- |
|  | **Mean±SD** | **Range** | **Mean±SD** | **Range** | **Mean±SD** | **Range** |
| **Metric character** | |  |  |  |  |  |
| **SVL*** | 74.6±3.5 | (66.0–80.9) | 63.0±2.4 | (59.1–67.5) | 90.5±1.5 | (88.8–92.7) |
| **Character ratios (%)** | |  |  |  |  |  |
| **RHL*** | 23.9±0.5 | (23.2–25.2) | 25.6±0.9 | (23.9–26.9) | 25.7±1.5 | (23.8–28.0) |
| **RHW**** | 16.6±0.7 | (15.3–18.1) | 17.0±0.7 | (15.7–18.4) | 19.5±0.5 | (18.7–20.2) |
| **RMXHW**** | 17.8±0.7 | (16.3–19.6) | 18.3±0.4 | (17.2–18.8) | 20.6±0.2 | (20.3–20.9) |
| **RLJL**** | 12.9±0.7 | (11.4–14.6) | 14.0±0.7 | (12.8–14.9) | 15.0±0.5 | (14.3–15.4) |
| **RSL**** | 6.7±0.3 | (6.3–7.4) | 6.6±0.2 | (6.1–6.9) | 7.5±0.1 | (7.4–7.6) |
| **RIND*** | 6.5±0.2 | (5.9–7.2) | 5.6±0.4 | (5.0–6.6) | 7.3±0.5 | (7.0–8.0) |
| **RIOD** | 5.8±0.2 | (5.4–6.2) | 5.6±0.1 | (5.4–5.8) | 6.6±0.2 | (6.3–6.9) |
| **RUEW*** | 3.3±0.1 | (3.2–3.6) | 3.5±0.1 | (3.4–3.7) | 3.4±0.2 | (3.2–3.6) |
| **RUEL** | 5.4±0.2 | (5.0–5.7) | 5.6±0.2 | (5.1–5.9) | 5.4±0.2 | (5.2–5.7) |
| **ROL*** | 2.9±0.1 | (2.6–3.2) | 3.5±0.2 | (3.0–3.9) | 3.4±0.1 | (3.3–3.5) |
| **RAGD** | 52.5±1.4 | (50.2–55.1) | 52.1±1.2 | (50.0–53.7) | 51.1±0.3 | (50.7–51.6) |
| **RTRL*** | 77.2±0.9 | (74.5–79.8) | 74.3±0.6 | (73.5–75.3) | 74.9±0.9 | (73.8–76.2) |
| **RTAL*** | 78.6±3.3 | (71.6–85.0) | 70.7±1.7 | (68.0–75.1) | 79.4±4.8 | (75.1–86.7) |
| **RBTAW** | 11.5±0.5 | (10.1–12.9) | 11.1±0.5 | (10.2–12.0) | 12.9±0.3 | (12.5–13.3) |
| **RMTAW*** | 9.0±0.4 | (7.8–10.0) | 7.7±0.6 | (6.4–8.6) | 9.7±0.6 | (9.0–10.7) |
| **RMXTAH** | 12.3±1.2 | (9.5–14.7) | 11.9±1.1 | (9.7–13.2) | 13.9±1.6 | (12.6–16.3) |
| **RMTAH** | 10.9±0.9 | (8.8–13.2) | 10.3±0.9 | (9.0–12.0) | 11.9±0.9 | (10.6–13.3) |
| **RFLL** | 23.6±0.9 | (21.5–25.6) | 22.9±1.1 | (20.5–24.7) | 23.1±0.3 | (22.8–23.5) |
| **RHLL*** | 28.7±0.7 | (26.6–30.1) | 27.4±0.6 | (26.4–28.2) | 27.7±0.6 | (26.8–28.4) |
| **R2FL** | 4.4±0.3 | (3.3–5.0) | 4.6±0.3 | (3.9–5.0) | 4.1±0.2 | (3.8–4.4) |
| **R3FL** | 3.8±0.4 | (2.7–4.6) | 3.7±0.1 | (3.5–4.0) | 3.7±0.1 | (3.6–3.8) |
| **R3TL** | 6.3±0.2 | (5.8–6.8) | 6.2±0.4 | (5.5–6.6) | 6.0±0.2 | (5.7–6.3) |
| **R5TL** | 2.2±0.3 | (1.6–2.7) | 1.9±0.4 | (1.4–3.2) | 2.4±0.2 | (2.3–2.7) |
| **RVTW*** | 5.9±0.2 | (5.5–6.3) | 5.5±0.1 | (5.3–5.7) | 6.7±0.1 | (6.7–6.8) |
| **RVTL*** | 4.9±0.2 | (4.3–5.3) | 7.1±0.1 | (7.0–7.2) | 7.0±0.1 | (6.8–7.1) |
| **VTW/VTL*** | 119.5±4.5 | (112.6–131.4) | 78.1±1.8 | (75.6–80.9) | 103.4±1.4 | (101.3–105.5) |
| **Meristic characters** | |  |  |  |  |  |
| **UJTN*** | 77.5±3.8 | (71–88) | 52.9±1.9 | (49–57) | 86.7±2.9 | (83–91) |
| **LJTN*** | 68.4±3.7 | (60–81) | 42.6±2.7 | (38–47) | 79.3±2.2 | (76–82) |
| **VTN** | 55.9±5.2 | (43–66) | 51.1±2.3 | (44–56) | 68.0±2.7 | (64–71) |
| **CGN** | 12.8±0.3 | (12–13) | 13.0±0.0 | (13–13) | 13.0±0.0 | (13–13) |
| **LON** | -1.3±0.5 | (-2.0 – -0.5) | -1.0±0.4 | (-2.0 – -0.5) | -2.2±0.6 | (-3.0 – -1.5) |
