# Supplementary material for: An integrative taxonomic analysis reveals a new species of lotic Hynobius salamander from Japan
Source: PeerJ. 2018 Jun 21;6:e5084. doi: 10.7717/peerj.5084 (PMC6015758; doi:10.7717/peerj.5084)
Supplement: Supplemental Information 8 [file peerj-06-5084-s008.docx]

| **Character** | **Factor 1** | **Factor 2** | **Factor 3** |
| --- | --- | --- | --- |
| **RHL** | 0.00% | 8.33% | 0.41% |
| **RHW** | 3.07% | 7.20% | 0.02% |
| **RMXHW** | 3.25% | 7.93% | 0.27% |
| **RLJL** | 0.46% | 5.52% | 7.20% |
| **RSL** | 6.23% | 1.16% | 0.75% |
| **RIND** | 8.36% | 0.88% | 0.84% |
| **RIOD** | 6.06% | 0.18% | 4.46% |
| **RUEW** | 0.07% | 3.52% | 4.26% |
| **RUEL** | 0.05% | 2.95% | 4.05% |
| **ROL** | 1.15% | 6.27% | 5.15% |
| **RAGD** | 0.56% | 4.12% | 0.15% |
| **RTRL** | 0.33% | 9.99% | 0.04% |
| **RTAL** | 6.06% | 0.42% | 4.55% |
| **RBTAW** | 5.35% | 2.10% | 1.95% |
| **RMTAW** | 8.64% | 0.06% | 0.80% |
| **RMXTAH** | 3.10% | 1.68% | 15.22% |
| **RMTAH** | 4.96% | 1.13% | 9.74% |
| **RFLL** | 1.85% | 0.27% | 1.88% |
| **RHLL** | 0.90% | 3.22% | 2.54% |
| **R2FL** | 0.00% | 0.64% | 1.57% |
| **R3FL** | 0.46% | 0.58% | 0.17% |
| **R3TL** | 0.51% | 1.47% | 0.10% |
| **R5TL** | 2.75% | 0.93% | 0.01% |
| **RVTW** | 7.65% | 0.24% | 0.00% |
| **RVTL** | 1.14% | 11.23% | 0.83% |
| **VTW/VTL** | 3.84% | 2.12% | 0.78% |
| **MTAW/MXTAH** | 1.12% | 3.14% | 13.60% |
| **MTAW/MTAH** | 0.33% | 2.87% | 9.44% |
| **UJTN** | 6.56% | 3.93% | 0.02% |
| **LJTN** | 8.43% | 2.64% | 0.11% |
| **VTN** | 3.33% | 0.00% | 7.41% |
| **CGN** | 3.41% | 3.27% | 1.69% |
