# Supplementary material for: An integrative taxonomic analysis reveals a new species of lotic Hynobius salamander from Japan
Source: PeerJ. 2018 Jun 21;6:e5084. doi: 10.7717/peerj.5084 (PMC6015758; doi:10.7717/peerj.5084)
Supplement: Supplemental Information 9 — For character abbreviations see Supplemental Information 4. 1–Character or character ratio to SVL exhibits sexual dimorphism (see Materials and methods; p<0.05); 2–denotes regenerated tail. Paired meristic characters (TN, VTN) are given in right/left order. Male holotype (ZMMU A-5862) marked in bold and with star (*). [file peerj-06-5084-s009.docx]

|  | **Specimen ID** | **Sex** | **SVL¹** | **HL¹** | **HW¹** | **MXHW¹** | **LJL** | **SL** | **IND** | **IOD** | **UEW** | **UEL** | **OL** | **AGD¹** | **TRL¹** | **TAL¹** | **BTAW¹** | **MTAW¹** |
| --- | --- | --- | --- | --- | --- | --- | --- | --- | --- | --- | --- | --- | --- | --- | --- | --- | --- | --- |
| 1 | YCM-RA-581 | m | 79.9 | 18.6 | 12.6 | 14.0 | 9.6 | 5.3 | 5.0 | 4.5 | 2.6 | 4.1 | 2.1 | 42.1 | 61.5 | 67.9 | 9.2 | 7.7 |
| 2 | YCM-RA-582 | m | 80.8 | 18.8 | 13.6 | 14.6 | 10.2 | 5.1 | 5.1 | 4.9 | 2.6 | 4.2 | 2.3 | 42.7 | 62.9 | 68.5 | 10.0 | 8.1 |
| 3 | YCM-RA-583 | m | 73.0 | 17.1 | 12.0 | 13.3 | 9.2 | 4.6 | 4.4 | 4.0 | 2.3 | 3.7 | 2.1 | 36.6 | 54.3 | 59.0 | 8.2 | 6.6 |
| 4 | YCM-RA-584 | m | 80.9 | 18.9 | 13.6 | 14.0 | 9.9 | 5.1 | 5.0 | 4.4 | 2.6 | 4.0 | 2.4 | 41.5 | 63.0 | 63.5 | 9.4 | 7.2 |
| 5 | YCM-RA-585 | m | 77.3 | 18.0 | 12.3 | 13.6 | 8.9 | 5.0 | 5.0 | 4.2 | 2.5 | 4.0 | 2.1 | 40.3 | 59.3 | 61.0 | 9.0 | 6.9 |
| 6 | ZMMU A-5851 | m | 78.9 | 18.5 | 14.0 | 14.3 | 10.2 | 5.8 | 4.7 | 4.3 | 2.8 | 4.3 | 2.3 | 43.5 | 60.5 | 58.2 | 10.2 | 7.3 |
| 7 | ZMMU A-5852 | m | 70.3 | 16.7 | 12.2 | 13.3 | 9.2 | 5.1 | 5.1 | 4.2 | 2.4 | 3.7 | 1.9 | 35.9 | 53.3 | 55.9² | 8.5 | 6.1 |
| 8 | ZMMU A-5853 | m | 70.4 | 17.0 | 11.1 | 13.0 | 8.9 | 5.1 | 4.5 | 3.9 | 2.2 | 3.8 | 2.0 | 35.9 | 54.0 | 58.6 | 8.4 | 6.2 |
| 9 | ZMMU A-5854 | m | 75.1 | 17.5 | 12.2 | 13.5 | 8.6 | 5.1 | 5.0 | 4.4 | 2.6 | 4.2 | 2.2 | 40.6 | 58.5 | 57.9 | 8.6 | 6.5 |
| 10 | **ZMMU A-5862*** | m | 69.4 | 17.5 | 12.2 | 13.2 | 9.7 | 4.4 | 4.4 | 4.2 | 2.4 | 3.8 | 2.2 | 35.9 | 52.9 | 58.4 | 8.2 | 6.3 |
| 11 | ZMMU A-5863 | m | 78.5 | 18.9 | 12.9 | 13.2 | 10.4 | 5.3 | 5.3 | 4.4 | 2.5 | 4.2 | 2.2 | 43.2 | 62.6 | 56.5² | 7.9 | 6.1 |
| 12 | ZMMU A-5864 | m | 73.4 | 18.2 | 12.9 | 13.4 | 10.1 | 5.1 | 4.9 | 4.1 | 2.5 | 3.9 | 2.1 | 40.4 | 58.0 | 57.6 | 7.9 | 6.9 |
| 13 | ZMMU A-5865 | m | 66.0 | 16.1 | 11.9 | 12.9 | 9.0 | 4.5 | 4.5 | 4.0 | 2.2 | 3.7 | 2.0 | 33.3 | 50.2 | 54.0 | 7.3 | 6.3 |
| 14 | ZMMU A-5867 | m | 72.4 | 17.3 | 11.9 | 13.0 | 9.8 | 5.2 | 5.0 | 4.2 | 2.4 | 3.9 | 2.2 | 37.1 | 55.8 | 54.4 | 8.8 | 6.8 |
| 15 | ZMMU A-5868 | m | 75.1 | 17.9 | 11.5 | 12.8 | 9.2 | 5.0 | 4.9 | 4.6 | 2.6 | 4.3 | 2.3 | 40.0 | 58.5 | 53.8 | 8.8 | 6.9 |
| 16 | ZMMU A-5869 | m | 76.6 | 18.3 | 11.7 | 12.6 | 10.5 | 5.1 | 5.0 | 4.5 | 2.7 | 4.2 | 2.2 | 39.5 | 60.0 | 57.6 | 8.9 | 7.4 |
| 17 | ZMMU A-5870 | m | 74.4 | 18.5 | 12.4 | 12.1 | 10.9 | 5.1 | 4.9 | 4.6 | 2.4 | 4.0 | 2.3 | 40.5 | 57.5 | 57.2 | 8.0 | 5.9 |
| 18 | ZMMU A-5871 | m | 69.9 | 17.0 | 11.2 | 12.2 | 9.2 | 4.5 | 4.6 | 4.3 | 2.5 | 4.0 | 2.2 | 36.5 | 54.2 | 53.9 | 7.5 | 6.1 |
|  | **Males Mean±SD** |  | **74.6±3.5** | **17.8±0.7** | **12.3±0.6** | **13.3±0.5** | **9.6±0.6** | **5.0±0.2** | **4.8±0.2** | **4.3±0.2** | **2.5±0.1** | **4.0±0.2** | **2.2±0.1** | **39.2±2.6** | **57.6±3.2** | **58.5±3.0** | **8.6±0.6** | **6.7±0.5** |
|  | **Range** |  | **(66.0–80.9)** | **(16.1–18.9)** | **(11.1–14.0)** | **(12.1–14.6)** | **(8.6–10.9)** | **(4.4–5.8)** | **(4.4–5.3)** | **(3.9–4.9)** | **(2.2–2.8)** | **(3.7–4.3)** | **(1.9–2.4)** | **(33.3–43.5)** | **(50.2–63.0)** | **(53.8–68.5)** | **(7.3–10.2)** | **(5.9–8.1)** |
| 19 | YCM-RA-586 | f | 82.5 | 18.8 | 12.7 | 14.3 | 9.5 | 5.0 | 5.1 | 5.0 | 2.6 | 4.4 | 2.5 | 45.9 | 64.8 | 62.2 | 9.1 | 6.3 |
| 20 | YCM-RA-587 | f | 76.6 | 17.9 | 12.1 | 13.4 | 9.2 | 5.3 | 4.9 | 4.7 | 2.6 | 4.1 | 2.2 | 43.6 | 61.1 | 57.4 | 8.5 | 6.9 |
| 21 | YCM-RA-588 | f | 77.9 | 18.4 | 12.0 | 12.6 | 9.0 | 4.9 | 5.1 | 4.5 | 2.6 | 4.0 | 2.3 | 43.5 | 60.0 | 58.5 | 8.6 | 6.6 |
| 22 | ZMMU A-5858 | f | 76.6 | 18.1 | 11.6 | 13.2 | 9.7 | 5.1 | 4.9 | 4.5 | 2.5 | 4.1 | 2.4 | 43.0 | 60.1 | 58.2 | 8.2 | 6.5 |
| 23 | ZMMU A-5859 | f | 76.2 | 17.8 | 12.3 | 13.4 | 9.0 | 5.1 | 4.7 | 4.6 | 2.5 | 4.2 | 2.4 | 42.1 | 60.4 | 54.9² | 8.1 | 6.3 |
| 24 | ZMMU A-5860 | f | 79.3 | 18.4 | 11.9 | 12.7 | 10.2 | 5.0 | 5.1 | 4.7 | 2.6 | 3.9 | 2.3 | 46.4 | 63.4 | 55.2 | 8.1 | 6.2 |
| 25 | ZMMU A-5861 | f | 78.6 | 18.6 | 13.5 | 13.8 | 10.3 | 5.3 | 5.1 | 4.5 | 2.7 | 4.0 | 2.4 | 44.6 | 61.6 | 55.8² | 8.5 | 6.3 |
| 26 | ZMMU A-5866 | f | 79.2 | 18.6 | 12.8 | 13.5 | 10.0 | 5.3 | 5.2 | 4.6 | 2.7 | 4.1 | 2.3 | 43.0 | 61.2 | 58.3 | 7.3 | 6.1 |
|  | **Females Mean±SD** |  | **78.4±1.5** | **18.3±0.3** | **12.3±0.5** | **13.3±0.4** | **9.6±0.4** | **5.1±0.1** | **5.0±0.2** | **4.6±0.1** | **2.6±2.7** | **4.1±0.1** | **2.4±0.1** | **44.0±1.2** | **61.6±1.3** | **57.6±1.7** | **8.3±0.4** | **6.4±0.2** |
|  | **Range** |  | **(76.2–82.5)** | **(17.8–18.8)** | **(11.6–13.5)** | **(12.6–14.3)** | **(9.0–10.3)** | **(4.9–5.3)** | **(4.7–5.2)** | **(4.5–5.0)** | **(2.5–2.7)** | **(3.9–4.4)** | **(2.2–2.5)** | **(42.1–46.4)** | **(60.0–64.8)** | **(54.9–62.2)** | **(7.3–9.1)** | **(6.1–6.9)** |
|  | **Total Mean±SD** |  | **75.7±3.4** | **18.0±0.6** | **12.3±0.6** | **13.3±0.5** | **9.6±0.5** | **5.1±0.2** | **4.9±0.2** | **4.4±0.2** | **2.5±0.1** | **4.0±0.2** | **2.2±0.1** | **40.7±2.8** | **58.8±3.1** | **58.2±2.6** | **8.5±0.5** | **6.6±0.4** |
|  | **Range** |  | **(66.0–82.5)** | **(16.1–18.9)** | **(11.1–14.0)** | **(12.1–14.6)** | **(8.6–10.9)** | **(4.4–5.8)** | **(4.4–5.3)** | **(3.9–5.0)** | **(2.2–2.8)** | **(3.7–4.4)** | **(1.9–2.5)** | **(33.3–46.4)** | **(50.2–64.8)** | **(53.8–68.5)** | **(7.3–10.2)** | **(5.9–8.1)** |

**(Continues on the next page)**

**Table 5. (Continued)**

|  | **Specimen ID** | **MXTAH** | **MTAH¹** | **FLL¹** | **HLL** | **2FL** | **3FL** | **3TL** | **5TL** | **VTW** | **VTL** | **UJTN** | **LJTN** | **TN (R/L)** | **CGN** | **LON** | **VTN** | **VTN (R/L)** |
| --- | --- | --- | --- | --- | --- | --- | --- | --- | --- | --- | --- | --- | --- | --- | --- | --- | --- | --- |
| 1 | YCM-RA-581 | 11.8 | 9.6 | 18.0 | 22.8 | 3.5 | 3.2 | 4.9 | 1.9 | 4.8 | 3.7 | 71 | 65 | 5/5 | 13 | -1.5 | 47 | (24/23) |
| 2 | YCM-RA-582 | 11.0 | 9.2 | 18.2 | 21.5 | 3.7 | 3.1 | 5.0 | 1.8 | 4.9 | 4.0 | 73 | 73 | 4/5 | 13 | -1.5 | 61 | (30/31) |
| 3 | YCM-RA-583 | 9.9 | 7.1 | 16.8 | 20.0 | 3.2 | 2.6 | 4.5 | 2.0 | 4.1 | 3.5 | 88 | 67 | 5/5 | 12 | -0.5 | 57 | (30/27) |
| 4 | YCM-RA-584 | 11.0 | 9.1 | 18.1 | 23.4 | 3.5 | 2.2 | 5.1 | 1.5 | 4.7 | 3.9 | 77 | 60 | 5/5 | 12 | -1.5 | 59 | (29/30) |
| 5 | YCM-RA-585 | 9.9 | 8.9 | 19.2 | 22.2 | 3.4 | 3.2 | 5.3 | 1.9 | 4.3 | 3.6 | 78 | 64 | 5/5 | 13 | -1.5 | 58 | (28/30) |
| 6 | ZMMU A-5851 | 10.4 | 9.3 | 17.0 | 21.0 | 2.6 | 2.4 | 5.0 | 1.6 | 5.0 | 4.2 | 77 | 69 | 4/5 | 13 | -1.5 | 50 | (24/26) |
| 7 | ZMMU A-5852 | 8.7 | 7.8 | 16.8 | 20.2 | 3.2 | 3.0 | 4.4 | 1.8 | 4.5 | 3.6 | 71 | 67 | 5/5 | 12 | -0.5 | 49 | (24/25) |
| 8 | ZMMU A-5853 | 9.4 | 7.9 | 16.5 | 20.1 | 3.4 | 2.9 | 4.1 | 1.2 | 3.9 | 3.3 | 72 | 64 | 5/5 | 13 | -0.5 | 43 | (21/22) |
| 9 | ZMMU A-5854 | 9.2 | 8.2 | 18.2 | 21.9 | 3.2 | 3.0 | 4.6 | 1.5 | 4.4 | 3.9 | 81 | 68 | 5/5 | 13 | -1 | 57 | (28/29) |
| 10 | **ZMMU A-5862*** | 10.0 | 9.2 | 17.0 | 20.2 | 3.1 | 3.2 | 4.6 | 1.5 | 4.1 | 3.6 | 73 | 70 | 5/5 | 13 | -1 | 53 | (27/26) |
| 11 | ZMMU A-5863 | 8.5 | 8.0 | 17.5 | 22.1 | 3.6 | 3.2 | 5.0 | 1.6 | 4.4 | 3.8 | 82 | 70 | 5/5 | 13 | -1.5 | 61 | (31/30) |
| 12 | ZMMU A-5864 | 8.6 | 8.6 | 17.1 | 21.5 | 3.6 | 3.2 | 5.0 | 1.9 | 4.5 | 3.9 | 79 | 81 | 5/5 | 13 | -1 | 50 | (27/23) |
| 13 | ZMMU A-5865 | 8.1 | 7.9 | 16.9 | 19.2 | 3.2 | 2.3 | 4.3 | 1.7 | 3.9 | 3.4 | 75 | 74 | 5/5 | 13 | -0.5 | 61 | (31/30) |
| 14 | ZMMU A-5867 | 8.0 | 7.1 | 16.9 | 21.8 | 3.6 | 2.9 | 4.6 | 1.5 | 4.3 | 3.7 | 76 | 61 | 5/5 | 13 | -1 | 53 | (27/26) |
| 15 | ZMMU A-5868 | 8.5 | 7.4 | 18.3 | 21.9 | 3.4 | 2.7 | 4.9 | 1.4 | 4.3 | 3.8 | 78 | 66 | 5/5 | 13 | -0.5 | 56 | (25/31) |
| 16 | ZMMU A-5869 | 8.1 | 7.6 | 18.9 | 22.6 | 3.1 | 2.5 | 5.0 | 1.9 | 4.4 | 3.3 | 88 | 72 | 5/5 | 13 | -1 | 60 | (30/30) |
| 17 | ZMMU A-5870 | 7.1 | 6.6 | 18.0 | 22.1 | 3.1 | 2.4 | 4.6 | 1.2 | 4.1 | 3.4 | 80 | 69 | 5/5 | 13 | -1.5 | 66 | (30/36) |
| 18 | ZMMU A-5871 | 7.2 | 7.0 | 17.0 | 20.6 | 3.3 | 2.5 | 4.5 | 1.2 | 4.1 | 3.4 | 76 | 71 | 5/5 | 12 | -0.5 | 66 | (32/34) |
|  | **Males Mean±SD** | **9.2±1.1** | **8.1±0.8** | **17.6±0.7** | **21.4±0.9** | **3.3±0.2** | **2.8±0.3** | **4.7±0.3** | **1.6±0.2** | **4.4±0.2** | **3.7±0.2** | **77.5±3.8** | **68.4±3.7** | **4.9±0.1** | **12.8±0.3** | **-1.0±0.4** | **55.9±5.2** |  |
|  | **Range** | **(7.1–11.8)** | **(6.6–9.6)** | **(16.5–19.2)** | **(19.2–23.4)** | **(2.6–3.7)** | **(2.2–3.2)** | **(4.1–5.3)** | **(1.2–2.0)** | **(3.9–5.0)** | **(3.3–4.2)** | **(71–88)** | **(60–81)** | **(4.5–5.0)** | **(12–13)** | **(-1.5 – -0.5)** | **(43–66)** |  |
| 19 | YCM-RA-586 | 9.6 | 7.9 | 17.9 | 22.2 | 3.5 | 2.9 | 5.1 | 2.2 | 4.5 | 3.8 | 80 | 71 | 5/5 | 13 | -2 | 59 | (29/30) |
| 20 | YCM-RA-587 | 9.0 | 7.8 | 17.3 | 22.0 | 3.6 | 3.0 | 4.8 | 2.1 | 4.6 | 3.5 | 76 | 64 | 5/5 | 13 | -2 | 62 | (32/30) |
| 21 | YCM-RA-588 | 9.9 | 8.3 | 17.4 | 22.0 | 3.3 | 2.9 | 5.4 | 2.2 | 4.6 | 4.0 | 80 | 61 | 5/5 | 13 | -2 | 44 | (21/23) |
| 22 | ZMMU A-5858 | 8.5 | 8.4 | 16.9 | 21.1 | 3.5 | 3.2 | 4.8 | 1.7 | 4.6 | 3.5 | 86 | 68 | 5/5 | 13 | -2 | 59 | (27/32) |
| 23 | ZMMU A-5859 | 9.3 | 7.5 | 16.3 | 22.6 | 3.5 | 3.2 | 4.6 | 1.4 | 4.1 | 3.3 | 71 | 66 | 5/5 | 13 | -2 | 51 | (26/25) |
| 24 | ZMMU A-5860 | 8.3 | 6.9 | 17.3 | 22.1 | 3.4 | 3.3 | 4.7 | 1.6 | 4.8 | 4.0 | 72 | 54 | 5/5 | 13 | -2 | 50 | (24/26) |
| 25 | ZMMU A-5861 | 8.2 | 7.0 | 18.9 | 22.7 | 3.8 | 3.3 | 4.9 | 2.2 | 4.4 | 3.8 | 82 | 70 | 5/5 | 13 | -1.5 | 60 | (29/31) |
| 26 | ZMMU A-5866 | 9.5 | 7.7 | 18.8 | 22.7 | 3.6 | 3.6 | 5.6 | 2.1 | 4.6 | 3.9 | 78 | 69 | 5/5 | 13 | -1.5 | 58 | (28/30) |
|  | **Females Mean±SD** | **9.0±0.5** | **7.7±0.4** | **17.6±0.7** | **22.2±0.4** | **3.5±0.1** | **3.2±0.2** | **5.0±0.3** | **1.9±0.3** | **4.5±0.2** | **3.7±0.2** | **78.1±3.9** | **65.4±4.3** | **5.0±0.0** | **13.0±0.0** | **-1.9±0.2** | **55.4±5.3** |  |
|  | **Range** | **(8.2–9.9)** | **(6.9–8.4)** | **(16.3–18.9)** | **(21.1–22.7)** | **(3.3–3.8)** | **(2.9–3.6)** | **(4.6–5.6)** | **(1.4–2.2)** | **(4.1–4.8)** | **(3.3–4.0)** | **(71–86)** | **(54–71)** | **(5.0–5.0)** | **(13–13)** | **(-2 – -1.5)** | **(44–62)** |  |
|  | **Total Mean±SD** | **9.1±0.9** | **8.0±0.7** | **17.6±0.7** | **21.6±0.8** | **3.4±0.2** | **2.9±0.3** | **4.8±0.3** | **1.7±0.3** | **4.4±0.2** | **3.7±0.2** | **77.7±3.8** | **67.5±3.9** | **5.0±0.1** | **12.8±0.3** | **-1.3±0.5** | **55.8±5.2** |  |
|  | **Range** | **(7.1–11.8)** | **(6.6–9.6)** | **(16.3–19.2)** | **(19.2–23.4)** | **(2.6–3.8)** | **(2.2–3.6)** | **(4.1–5.6)** | **(1.2–2.2)** | **(3.9–5.0)** | **(3.3–4.2)** | **(71–88)** | **(54–81)** | **(4.5–5.0)** | **(12–13)** | **(-2.0 – -0.5)** | **(43–66)** |  |
