# Supplementary material for: An integrative taxonomic analysis reveals a new species of lotic Hynobius salamander from Japan
Source: PeerJ. 2018 Jun 21;6:e5084. doi: 10.7717/peerj.5084 (PMC6015758; doi:10.7717/peerj.5084)
Supplement: Supplemental Information 10 — For character abbreviations see Supplemental Information 4. [file peerj-06-5084-s010.docx]

| **Specimen** | **EGN** | **EGD** | **ESL** | **ESW** | **WTL** |
| --- | --- | --- | --- | --- | --- |
| ZMMU A-5879 | 15 | 4.9±0.2 | 54.6 | 18.9 | 22.6 |
| ZMMU A-5880 | 16 | 5.2±0.1 | 44.1 | 18.4 | 19.9 |
| ZMMU A-5881 | 12 | 5.4±0.1 | 60.5 | 26.2 | 40.1 |
| ZMMU A-5882 | 16 | 5.6±0.2 | 61.5 | 21.1 | 31.0 |
| **Mean±SD** | **14.8±1.4** | **5.3±0.2** | **55.2±5.8** | **21.2±2.5** | **28.4±7.1** |
| **Range** | **(12–16)** | **(4.9–5.6)** | **(44.1–61.5)** | **(18.4–26.2)** | **(19.9–40.1)** |
