# Supplementary material for: An integrative taxonomic analysis reveals a new species of lotic Hynobius salamander from Japan
Source: PeerJ. 2018 Jun 21;6:e5084. doi: 10.7717/peerj.5084 (PMC6015758; doi:10.7717/peerj.5084)
Supplement: Supplemental Information 11 — For character abbreviations see Supplemental Information 4. Staging is estimated according to Akita (2001). [file peerj-06-5084-s011.docx]

| **Specimen** | **Stage** | **SVL** | **HL** | **HW** | **OL** | **AGD** | **TAL** | **FLL** | **HLL** | **MXTAH** |
| --- | --- | --- | --- | --- | --- | --- | --- | --- | --- | --- |
| ZMMU A-5872 | 56 | 24.2 | 8.1 | 7.1 | 1.4 | 13.3 | 22.1 | 7.4 | 6.7 | 7.6 |
| ZMMU A-5873 | 56 | 24.9 | 9.1 | 7.0 | 1.3 | 13.2 | 21.5 | 6.5 | 6.2 | 7.2 |
| ZMMU A-5874 | 56 | 27.5 | 10.1 | 7.2 | 1.4 | 15.6 | 26.5 | 7.4 | 6.5 | 7.3 |
| ZMMU A-5875 | 56 | 27.2 | 9.2 | 7.0 | 1.2 | 14.5 | 24.2 | 7.1 | 7.0 | 7.1 |
| ZMMU A-5876 | 56 | 21.9 | 8.6 | 6.0 | 1.3 | 11.5 | 21.2 | 6.0 | 4.6 | 6.4 |
| ZMMU A-5877 | 57 | 28.5 | 9.3 | 7.0 | 1.2 | 14.2 | 25.5 | 7.2 | 7.1 | 5.1 |
| ZMMU A-5878 | 57 | 26.6 | 8.3 | 7.0 | 1.4 | 15.6 | 25.2 | 6.6 | 6.3 | 7.3 |
| **Mean±SD** |  | **25.8±1.9** | **9.0±0.5** | **6.9±0.3** | **1.3±0.1** | **14.0±1.1** | **23.7±1.8** | **6.9±0.4** | **6.3±0.5** | **6.9±0.6** |
| **Range** |  | **(21.9–28.5)** | **(8.1–10.1)** | **(6.0–7.2)** | **(1.2–1.4)** | **(11.5–15.6)** | **(21.2–26.5)** | **(6.0–7.4)** | **(4.6–7.1)** | **(5.1–7.6)** |
