# Supplementary material for: An integrative taxonomic analysis reveals a new species of lotic Hynobius salamander from Japan
Source: PeerJ. 2018 Jun 21;6:e5084. doi: 10.7717/peerj.5084 (PMC6015758; doi:10.7717/peerj.5084)
Supplement: Supplemental Information 12 [file peerj-06-5084-s012.docx]

**Supplementary TEXT File 1.**

**Brief review on history of taxonomic and phylogenetic studies of *Hynobius kimurae* species complex.**

*Hynobius kimurae* was described by *Dunn (1923a)* from Mt. Hieizan eastwards from Kyoto based on a single female specimen. The species was, however, known to herpetologists long before its formal description and was often confused with *H. naevius* (Temminck & Schlegel, 1838) (*Okada, 1891*; *Stejneger, 1907*; *Abe, 1922*).

*Tago (1907)* reported on this species as a new salamander from Hida Mountains in Gifu prefecture, which he assigned to the genus *Salamandrella* Dybowski, 1870 based on presence of four toes in his specimen, however without providing a formal description*.*

Later, *Hatta (1914, 1921)* mentioned “*Hynobius luteopunctatus*” for Honshu but did not provide description for this name, which is considered as a *nomen nudum*; it was placed into synonymy of *H. kimurae* by *Sato (1943)*.

Well after publications by *Dunn (1923a,b)*, *Tago (1929)*, possibly being unaware of his work, proposed the name “*Pseudosalamandra hida*” for *H. kimurae* from Hida Mountains in Gifu Prefecture of central Honshu; this name is also considered as a *nomen nudum* (*Frost, 2018*). Soon after, in his monograph, *Tago (1931)* regarded this species as *Pseudosalamandra kimurai*.

*Sato (1943)* provided an extensive account on morphology, variation, distribution and ecology of this species (as *Hynobius kimurae*, following *Dunn, 1923b*), indicating its affinities with *H. naevius*. This superficial similarity led *Nakamura & Ueno (1963)* to consider Hida salamander as a subspecies of the latter as *H. naevius kimurai*.

However, consequent works demonstrated that these two species often occur in syntopy (*Kuramoto, 1969*; *Matsui, 1979, 1981*; *Okada et al., 1997*) and *H. kimurae* was reconsidered as a distinct species.

Recent mitochondrial DNA-based phylogenetic works indicate that *H. kimurae* is not closely related to *H. naevius* complex, but is suggested as a sister species to *H. boulengeri* (Thompson, 1912), a large-sized lotic salamander from mountains of Kii Peninsula in central Honshu (*Larson et al., 2003*; *Larson et al., 2003*; *Li et al., 2011*; *Zheng et al., 2012*; *Nishikawa & Matsui, 2014*), originally described in a separate genus *Pachypalaminus* Thompson, 1912. These two species form a distant lineage sister to all other *Hynobius* except *H. retardatus* Dunn, 1923.

*Weisrock et al. (2013)*, however, presented an alternative topology with *H. kimurae* forming a sister lineage with respect to *H. hirosei* from Shikoku, while *H. boulengeri* is grouped together with members of the *H. neavius* complex. Phylogenetic tree of *Weisrock et al. (2013)* was based on the same dataset as in *Larson et al., 2003*. The sequences of *H. kimurae* and *H. boulengeri* of *Weisrock et al. (2013)* were available in GenBank for download early before their paper was published; these sequences were used in a number of studies on *Hynobius* phylogeny, including *Li et al., 2011*; *Zheng et al., 2012*, all of which resulted in the different topology with *H. kimurae* and *H. boulengeri* s. stricto forming a clade. Thus, the discrepant topology presented in *Weisrock et al. (2013)* is likely erroneous, possibly based on mislabeling or another mistake during preparation of their manuscript.

**Supplementary TEXT File 2.**

**List of lotic *Hynobius* species and specimens used for comparisons.**

***Hynobius fossigenus* sp.nov.:** ZMMU A-5851-5854, A-5858-5871, 18 adult specimens from Hinode Mt., Hinode, Tokyo (holotype and 12 paratypes); YCM-RA-581-588, 8 adult specimens from Hinode Mt., Hinode, Tokyo (all paratypes); ZMMU A-5872-5873, 2 larval specimens from Kamisano, Nanbu, Yamanashi Pref.; ZMMU A-5874-5876, 3 larval specimens from Misawa, Toyone, Aichi Pref.; ZMMU A-5877-5878, 2 larval specimens from Kurakake, Hinohara, Tokyo; ZMMU A-5879-5882, 4 egg sacs from Hinode Mt., Hinode, Tokyo; ZMMU A-5883-5884, 2 subadult specimens from Ootaba, Okutama, Tokyo; ZMMU A-5885-5890, 6 adult specimens from Urayama, Chichibu, Saitama Pref.; ZMMU A-5891-5898, 8 adult specimens from Kamisano, Nanbu, Yamanashi Pref.; NSMT 3676, 1 adult specimen from Okusawa-dani, Hayakawa, Minamikoma, Yamanashi Pref.; NSMT 1056, 1 adult specimen from Mt. Gozen-yama, Okutama, Tokyo;

***H. boulengeri*:** ZMMU A-5842, 2 adult specimens from Ooto-San Mt., Kozagawa, Wakayama Pref.; NSMT 1156, 1 adult specimen from Ohno, Totsugawa-mura, Yoshino, Nara Pref.;

***H. hirosei*: N**SMT 1154, 1 adult specimen from Tengu-dake, Asahinomaru, Kamiyama, Tokushima Pref.; NSMT 3389, 3392, 2 adult specimens from Takashiro-yama, Tokushima Pref.;

***H. ikioi*:** NSMT 4363-4368, 6 adult specimens from Nagamata, Honzawa-mura, Kochi Pref.;

***H. kimurae* sensu stricto:** ZMMU A-5899-5910, 12 adult specimens from Kumogahata, Kyoto; NSMT 3713-3726, 13 adult specimens from Goka-mura, Naka-gun, Kyoto;

***H. naevius*:** NSMT 3734-3736, 4 adult specimens from Mt. Daisen, Tottori Pref.;

***H. osumiensis*:** NSMT 3664, 1 adult specimen from Mt. Inao-dake, Tashiro-cho, Osumi, Kagoshima Pref.;

***H. stejnegeri*:** NSMT 3890-3891, 3893-3895, 4536-4538, 8 adult specimens from Takashiro-yama, Tokushima Pref.

**Supplementary TEXT File 3.**

**Primers used for PCR and sequencing.**

**16S rRNA gene (following *Hedges, 1994*)**

16L-1 (forward) (5’-CTGACCGTGCAAAGGTAGCGTAATCACT-3’)

16H-1 (reverse) (5’-CTCCGGTCTGAACTCAGATCACGTAGG-3’)

**cyt b gene (following *Matsui et al., 2008*)**

HYD-Cytb-F1 (forward) (5’-CYAAYCCTAAAGCWGCAAAATA-3’)

HYD-Cytb-R1 (reverse) (5’-TARRAARTGAAATGCAAARAATCG-3’)

**RAG1 gene (following *Nishikawa et al., 2013*)**

*RAG1*_F_N1 (forward) (5’-RCAAGCCRAAYTCAGAGYTRTGCTGCAAGC-3’)

*RAG1*_R_N3 (reverse) (5’-TTCATTCTCATTATGGGCYTCARRTTCATCTTC -3’)

**Supplementary TEXT File 4.**

**Morphological characters examined in this study (following *Nishikawa et al., 2007*; *Nishikawa, 2008*; *Matsui et al., 2009*;** ***Poyarkov et al., 2012*).**

**A. Metric characters:**

1. snout-vent length measured from snout tip to anterior edge of cloaca (SVL) (***also recorded for larval specimens***);
2. head length from snout tip to gular fold (HL) (***also recorded for larval specimens***);
3. head width measured at the level of anterior edge of parotoid gland (HW) (***also recorded for larval specimens***);
4. maximal head width (MXHW);
5. lower jaw length mouth angle to tip of lower jaw (LJL);
6. snout length measured from snout tip to anterior corner of eye (SL);
7. internarial distance (IND);
8. interorbital distance (IOD);
9. width of upper eyelid (UEW);
10. length of upper eyelid (UEL);
11. orbit length (OL) (***also recorded for larval specimens***);
12. axilla-groin distance (AGD) (***also recorded for larval specimens***);
13. trunk length, measured from anterior tip of cloaca to gular fold (TRL);
14. tail length from tail tip to anterior tip of cloaca (TAL) (***also recorded for larval specimens***);
15. basal tail width, measured at the level of anterior edge of cloaca (BTAW);
16. tail width measured at middle of its length (MTAW);
17. maximum tail height (MXTAH) (***also recorded for larval specimens***);
18. tail height measured at middle of its length (MTAH);
19. forelimb length, measured from forelimb base to tip of the longest finger (FLL) (***also recorded for larval specimens***);
20. hindlimb length, measured from hindlimb base to tip of the longest toe (HLL) (***also recorded for larval specimens***);
21. second finger length (2FL);
22. third finger length (3FL);
23. third toe length (3TL);
24. fifth toe length (5TL);
25. vomerine tooth series maximum width (VTW);
26. vomerine tooth series maximum length (VTL).

**B. Meristic characters included:**

1. upper jaw teeth number (UJTN);
2. lower jaw teeth number (LJTN);
3. vomerine teeth number (VTN; counted for right/left VTS);
4. toe number (TN);
5. costal groove number (CGN);
6. costal fold number between adpressed limbs (LON; minus and plus values correspond to separation and overlap, respectively).

**C. Additional morphometric characters used for holotype description:**

1. intercanthal distance (ICD);
2. chest width (CW);
3. orbitonarial distance (ON);
4. nostril-snout distance (NSD);
5. first finger length (1FL);
6. fourth finger length (4FL);
7. first toe length (1TL);
8. second toe length (2TL);
9. fourth toe length (4TL);
10. cloacal slit length (CSL).

**D. Egg sac morphology characters, measurements and counts:**

1. presence of the whiptail-like structure (WT);
2. number of eggs (per egg sac) (EGN);
3. average egg diameter (measured for 10 eggs per egg sac) (EGD);
4. maximum length of egg sac without whiptail structure on the free end (ESL);
5. egg sac width at its middle (ESW);
6. whiptail structure length (WTL).

**Supplementary TEXT File 5.**

**Additional morphological comparisons between *Hynobius fossigenus* sp. nov. and *H. kimurae* s. str.**

*Hynobius fossigenus* **sp. nov.** has comparatively shorter head (RHL; df=2; p=0.009); greater internarial distance (RIND; df=2; p=0.002), narrower upper eyelid (RUEW; df=2; p=0.0129), smaller orbit length (ROL; df=2; p=0.0001), greater trunk length (RTRL; df=2; p=0.0001) and tail length (specimens with regenerated tail excluded from analysis; RTAL; df=2; p=0.0004), greater width of tail in the middle (RMTAW; df=2; p=0.0004), longer hindlimbs (RHLL; df=2; p=0.0031), wider and shorter VTS (RVTW; df=2; p=0.0007; RVTL; df=2; p=0.0000), and generally shallower VTS (VTW/VTL; df=2; p=0.0001) than *H. kimurae* s. str.

Significant differences were found between number of teeth on upper and lower jaws, which was always greater in *Hynobius fossigenus* **sp. nov.**, the values did not overlap: UJTN 77.5±3.8 (71–88) (vs. 52.9±1.9; 49–57 in *H. kimurae* s. str.; df=2; p=0.0000), LJTN 68.4±3.7 (60–81) (vs. 42.6±2.7; 38–47 in *H. kimurae* s. str.; df=2; p=0.0000).

**References cited in the Supplementary Text Files:**

Abe Y. 1922. On Ambystomidae from Japan. *Zoological Magagine* 34:328–332 [in Japanese].

Dunn ER. 1923a. New species of *Hynobius* from Japan. *Proceedings of the California Academy of Sciences*, 4th Series 12:27–29.

Dunn ER. 1923b. The salamanders of the family Hynobiidae. *Proceedings of the American Academy of Arts and Sciences* 58:445–523.

Frost DR. 2018. *Amphibian Species of the World: an Online Reference*. Version 6.0. New York: American Museum of Natural History. *Available at http://research.amnh.org/herpetology/amphibia/index.html* (accessed 31 March 2018).

Hatta S. 1914. Zur Tiergeographie von Hokkaido. *Zoologischer Anzeiger* 43:27–36.

Hatta S. 1921. Some points on the Zoo-geography of Japan. *Journal of Geography*, Tokyo 33:654 [in Japanese].

Hedges SB. 1994. Molecular evidence for the origin of birds. *Proceedings of the National Academy of Sciences of the United States of America* 91:2621–2624.

Huang H, Lin B, Guo C, Tang Z, Wu Z. 2016. The complete mitochondrial genome of the *Hynobius maoershanensis* (Caudata: Hynobiidae). *Mitochondrial DNA Part A* 27:173–174.

Kuramoto M. 1969. Salamanders of western Japan. *Natural Study* 15:34–40 [in Japanese].

Larson A, Weisrock DW, Kozak KH. 2003. Phylogenetic systematics of salamanders (Amphibia: Urodela), a review. In: Sever DM, ed. *Reproductive Biology and Phylogeny of Urodela (Amphibia)*. New Hampshire: Science Publishers Inc, 31–108.

Lee BH, Kim JY, Song S, Hur JM, Cho JY, Park YC. 2011. The complete mitochondrial genome sequence of the Kori salamander *Hynobius yangi* (Caudata: Hynobiidae). *Mitochondrial DNA* 22:168–170.

Li J, Fu C, Lei G. 2011. Biogeographical consequences of Cenozoic tectonic events within East Asian margins: A case study of *Hynobius* biogeography. *PLoS One*, 6(6):e21506.

Matsui M, Misawa Y, Nishikawa K. 2009. Morphological Variation in a Japanese Salamander, *Hynobius kimurae* (Amphibia, Caudata). *Zoological Science* 26: 87–95.

Matsui M, Tominaga A, Hayashi T, Misawa Y, Tanabe S. 2007. Phylogenetic relationships and phylogeography of *Hynobius tokyoensis* (Amphibia: Caudata) using complete sequences of cytochrome b and control region genes of mitochondrial DNA. *Molecular Phylogenetics and Evolution* 44:204–216.

Matsui M, Yoshikawa N, Tominaga A, Sato T, Takenaka S, Tanabe S, Nishikawa K, Nakabayashi S. 2008. Phylogenetic relationships of two *Salamandrella* species as revealed by mitochondrial DNA and allozyme variation (Amphibia: Caudata: Hynobiidae). *Molecular phylogenetics and evolution* 48:84–93.

Matsui M. 1979. *Hynobius kimurae*. In: Sengoku S, ed. *Amphibians/Reptiles in Color*. Tokyo: Ie-no-Hikari-Kyokai, 106–107 [in Japanese].

Matsui M. 1981. *Hynobius kimurae*. In: Nature Conservation Society of Japan, ed. *Final Report of Reptiles and Amphibians Survey of the Second National Survey on the Natural Environment in Japan, 1978, Pt 2*. Tokyo: Nature Conservation Society of Japan, 151–158 [in Japanese].

Nakamura K, Ueno S. 1963. *Japanese Reptiles/Amphibians in Color*. Osaka: Hoikusha Publishers Co.,Ltd [in Japanese].

Nishikawa K, Khonsue W, Pomchote P, Matsui M. 2013. Two new species of *Tylototriton* from Thailand (Amphibia: Urodela: Salamandridae). *Zootaxa* 3737:261–279.

Nishikawa K, Matsui M, Tanabe S, Sato S. 2007. Morphological and allozymic variation in *Hynobius boulengeri* and *H. stejnegeri* (Amphibia: Urodela: Hynobiidae). *Zoological Science* 24:752–766.

Nishikawa K, Matsui M. 2014. Three new species of the salamander genus *Hynobius* (Amphibia, Urodela, Hynobiidae) from Kyushu, Japan. *Zootaxa* 3852:203–226.

Okada S, Utsunomiya T, Utsunomiya Y. 1997. On four species of salamanders from Mt. Omangi, Hiroshima Pref. *Hibakagaku* 179:1–7 [in Japanese].

Okada S. 1891. *Catalogue of the Vertebrated Animals of Japan*. Tokyo: Kinkodo [in Japanese].

Poyarkov NA, Che J, Min MS, Kuro-o M, Yan F, Li C, Iizuka K, Vieites DR. 2012. Review of the systematics, morphology and distribution of Asian Clawed Salamanders, genus *Onychodactylus* (Amphibia, Caudata: Hynobiidae), with the description of four new species. *Zootaxa* 3465:1–106.

Sato I. 1943. *Monograph of the Tailed Batrachians of Japan*. Osaka: Nippon Publisher [in Japanese].

Stejneger L. 1907. Herpetology of Japan and adjacent territory. *Bulletin of the United States National Museum* 58:xx + 577.

Tago K. 1907. Studies on the Urodela of Japan. II. *Zoological Magazine* 19:229–248 [in Japanese].

Tago K. 1929. Tailed batrachia of Japan. *Zoological Magazine* 41:430–432 [in Japanese] .

Tago K. 1931. *Newt and Salamander*. Tokyo: Maruzen [in Japanese].

Weisrock DW, Macey JR, Matsui M, Mulcahy DG, Papenfuss TJ. 2013. Molecular phylogenetic reconstruction of the endemic Asian salamander family Hynobiidae (Amphibia, Caudata). *Zootaxa* 3626:77–93.

Zhang P, Chen YQ, Zhou H, Liu YF, Wang XL, Papenfuss TJ, Wake DB, Qu LH. 2006. Phylogeny, evolution, and biogeography of Asiatic Salamanders (Hynobiidae). *Proceedings of the National Academy of Sciences of the United States of America* 103:7360–7365.

Zheng Y, Peng R, Murphy RW, Kuro-o M, Hu L, Zeg X. 2012. Matrilineal genealogy of *Hynobius* (Caudata: Hynobiidae) and a temporal perspective on varying levels of diversity among lineages of salamanders on the Japanese Islands. *Asian Herpetological Research* 3(4):288–302.
